# Functional characterization of *ent*-copalyl diphosphate synthase, kaurene synthase and kaurene oxidase in the *Salvia miltiorrhiza* gibberellin biosynthetic pathway

**DOI:** 10.1038/srep23057

**Published:** 2016-03-14

**Authors:** Ping Su, Yuru Tong, Qiqing Cheng, Yating Hu, Meng Zhang, Jian Yang, Zhongqiu Teng, Wei Gao, Luqi Huang

**Affiliations:** 1 School of Traditional Chinese Medicine, Capital Medical University, Beijing, China; 2 State Key Laboratory Breeding Base of Dao-di Herbs, National Resource Center for Chinese Materia Medica, China Academy of Chinese Medical Sciences, Beijing, China; 3 State Key Laboratory of Quality Research in Chinese Medicine, Macau University of Science and Technology, Avenida Wai Long, Taipa, Macau, China; 4 Department of Chemical and Biological Engineering, Chalmers University of Technology, Kemivägen 10, SE-41296 Göteborg, Sweden

## Abstract

*Salvia miltiorrhiza* Bunge is highly valued in traditional Chinese medicine for its roots and rhizomes. Its bioactive diterpenoid tanshinones have been reported to have many pharmaceutical activities, including antibacterial, anti-inflammatory, and anticancer properties. Previous studies found four different diterpenoid biosynthetic pathways from the universal diterpenoid precursor (*E*,*E*,*E*)-geranylgeranyl diphosphate (GGPP) in *S. miltiorrhiza*. Here, we describe the functional characterization of *ent*-copalyl diphosphate synthase (SmCPS_*ent*_), kaurene synthase (SmKS) and kaurene oxidase (SmKO) in the gibberellin (GA) biosynthetic pathway. SmCPS_*ent*_ catalyzes the cyclization of GGPP to *ent*-copalyl diphosphate (*ent*-CPP), which is converted to *ent*-kaurene by SmKS. Then, SmKO catalyzes the three-step oxidation of *ent*-kaurene to *ent*-kaurenoic acid. Our results show that the fused enzyme SmKS-SmCPS_*ent*_ increases *ent*-kaurene production by several fold compared with separate expression of SmCPS_*ent*_ and SmKS in yeast strains. In this study, we clarify the GA biosynthetic pathway from GGPP to *ent*-kaurenoic acid and provide a foundation for further characterization of the subsequent enzymes involved in this pathway. These insights may allow for better growth and the improved accumulation of bioactive tanshinones in *S. miltiorrhiza* through the regulation of the expression of these genes during developmental processes.

*Salvia miltiorrhiza* Bunge has been widely used in China (and to a lesser extent in Japan, the United States, and European countries) for the treatment of cardiovascular and cerebrovascular diseases. This medicinal herb exhibits anti-inflammatory, antioxidant and radical scavenging effects^1^,^2^. Tanshinone I, tanshinone IIA, cryptotanshinone and dihydrotanshinone I are the major diterpene quinones of the lipophilic constituents in Danshen and are responsible for much of its anti-inflammatory, antioxidant, antitumor and a variety of other activities^3^,^4^,^5^. Because these monomeric compounds have significant pharmacological activities, Danshen preparations are more frequently used in the clinic.

To accommodate the increasing need for clinical applications, researchers have deeply investigated the diterpenoid biosynthetic pathway to obtain the bioactive tanshinones directly using synthetic biology strategies in microbial cell factories. Previous works have indicated that at least four different diterpenoid biosynthetic pathways exist in *S. miltiorrhiza* (Fig. 1)^6^. Among them, the tanshinone biosynthetic pathway is uniquely initiated by a sequential pair of cyclization reactions catalyzed by SmCPS1 and SmKSL1 to produce abietane miltiradiene, which is a precursor of at least cryptotanshinone^7^,^8^. Then, SmCYP76AH1 catalyzes the turnover of miltiradiene to form ferruginol, thereby providing a solid foundation to elucidate the tanshinone biosynthetic pathway.

However, only two diterpene synthases (diTPSs) in the *S. miltiorrhiza* GA biosynthetic pathway have been reported to date, and the roles of GAs in *S. miltiorrhiza* root and rhizome development and the total yield of tanshinones per plant are less clear. GAs are formed from GGPP via a set of reactions catalyzed by different enzymes, including two consecutive diTPSs, cytochrome P450 (CYP) and 2-oxoglutarate-dependent dioxygenases (2ODDs) in plants^9^. As a group of plant-growth regulators, these GAs control different aspects of plant development, such as seed germination, stem elongation, flowering, fruit set and fruit development. Understanding GA biosynthesis will allow us to improve the tanshinone contents by regulating the expression of the genes involved in the *S. miltiorrhiza* GA biosynthetic pathway. Here, we cloned three genes (*SmCPS*_*ent*_, *SmKS* and *SmKO*) from *S. miltiorrhiza* hairy roots and then identified their functions by co-expressing them in *Saccharomyces cerevisiae*. Biochemical studies suggested that CPS and KS might interact with one another^10^; therefore, we constructed a fused SmCPS_*ent*_ and SmKS protein and showed that the production of *ent*-kaurene was significantly improved.

## Results

### Cloning and sequence analysis of *SmCPS*_*ent*_, *SmKS* and *SmKO* from *S. miltiorrhiza* hairy roots

**Figure 2:**
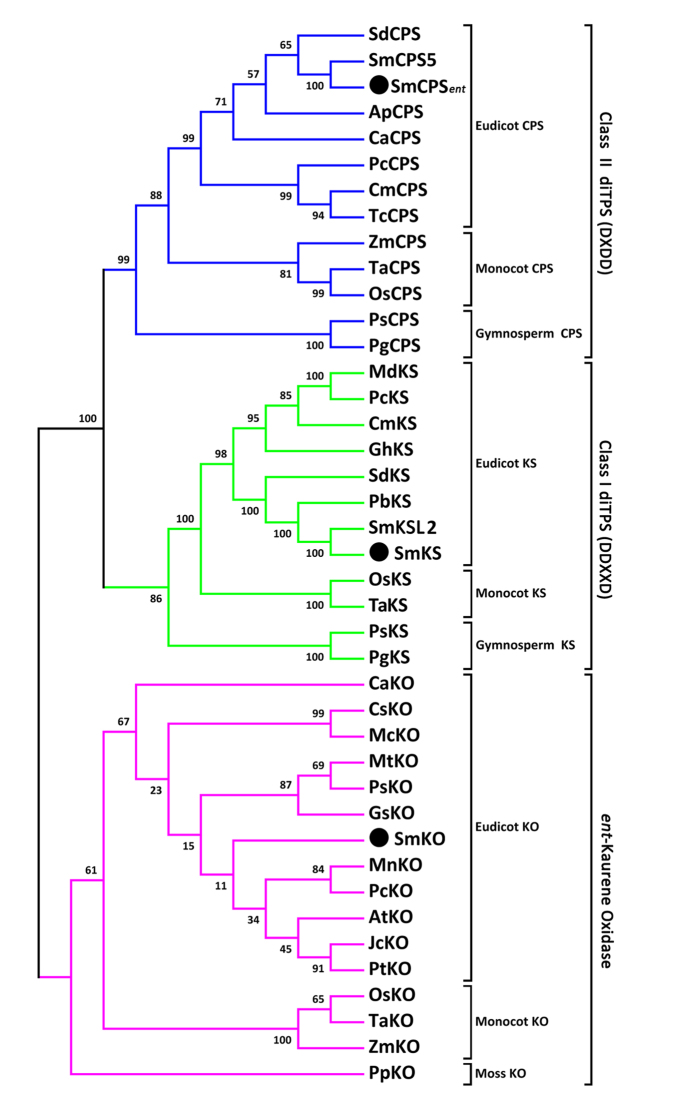
Phylogenetic tree of CPS, KS and KO from different species. The neighbor-joining phylogenetic trees were constructed using the bootstrap method in MEGA 5.1. The number of bootstrap replications was 1000. Descriptions of the three different types of synthases used in the phylogeny are listed in Supplementary Table 2.

**Figure 3:**
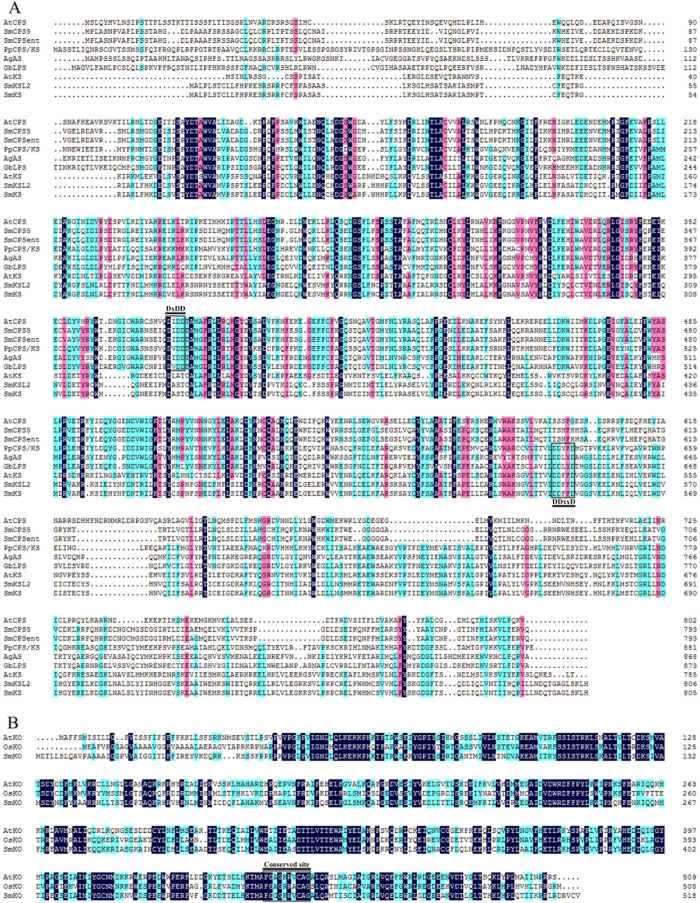
Alignment of plant CPS, KS and KO. The boxes represent the conserved regions. (**A**) AtCPS (Q38802), SmCPS5 (AHJ59324), SmCPS_*ent*_ (ALX18648), PpCPS/KS (BAF61135), AgAS (Q38710), GbLPS (Q947C4), AtKS (AAC39443), SmKSL2 (AHJ59325), SmKS (ALX18649). (**B**) AtKO (AAC39507), OsKO (AAT81230), SmKO (AJF93403).

The full-length *SmCPS*_*ent*_ and *SmKS* cDNAs were determined by 5′ RACE and 3′ RACE, and the corresponding cDNA sequences were submitted to the National Center for Biotechnology Information (Supplementary Fig. S1). The full-length *SmCPS*_*ent*_ cDNA (GenBank accession number KT934789) is 2413 nt and encodes a polypeptide of 793 amino acids. SmCPS_*ent*_ clusters most closely to SmCPS5 of *S*. *miltiorrhiza* f. alba and to SdCPS from *Scoparia dulcis* (Fig. 2). The first 21 N-terminal amino acids are rich in serine and threonine (19%), which is a common characteristic of transit peptides that target the diTPS to plastids^11^,^12^. This information was supported by our analysis using ChloroP 1.1 13. The amino acid sequence also contains a conserved DIDD motif (Fig. 3), which strongly suggests that SmCPS_*ent*_ can catalyze GGPP to CPP as a class II diTPS. The *SmKS* cDNA (GenBank accession number KT934790) is 2636 nt in length and encodes a predicted protein of 806 amino acid residues. At the protein level, the KS sequence from the hairy roots of *S*. *miltiorrhiza* exhibits 99% identity with the SmKSL2 from *S*. *miltiorrhiza* f. alba (Fig. 2). The first 27 N-terminal amino acids are rich in serine and threonine (22%), suggesting that SmKS is also localized in plastids. Its amino acid sequence contains a DDFFD motif but lacks the DxDD motif (Fig. 3), indicating that SmKS is a plant KS protein with monofunctional class I diTPS activity. The *SmKO* cDNA (GenBank accession number KJ606394) is 1930 nt in length and has an open reading frame (ORF) encoding 519 amino acid residues, containing a cytochrome P450 conserved site (amino acids 451–460, Fig. 3). The deduced amino acid sequence shows 64% and 66% identity with *AtKO* (*Arabidopsis thaliana*, AAC39507) and *PsKO* (*Pisum sativum*, AAP69988) (Fig. 2). The gene was identified as a multifunctional kaurene oxidase catalyzing three sequential oxidations (*ent*-kaurene to *ent*-kaurenoic acid) in the GA biosynthetic pathway^14^,^15^.

### Recombinant expression and functional characterization of SmCPS_*ent*_ and SmKS

**Figure 4:**
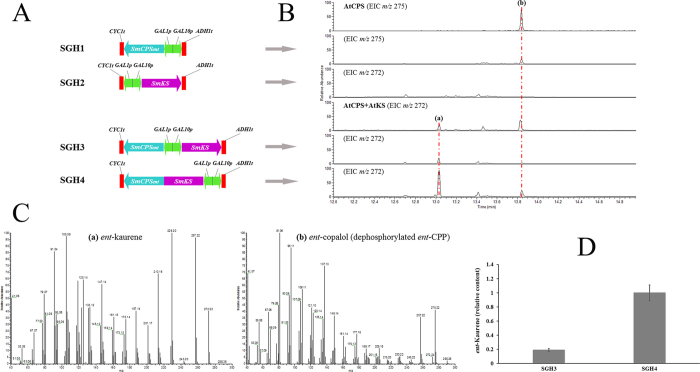
Extracts from yeast strain BY-T20 cultures expressing the *S. miltiorrhiza SmCPS*_*ent*_ or/and *SmKS* using GC-MS. (**A**) Construction of the recombinant plasmid in yeast strains. **BY-T20**: BY4742, *ΔTrp1*, *Trp1::HIS3-P*_*PGK1*_*-BTS1/ERG20-T*_*ADH1*_*-P*_*TDH3*_*-SaGGPS-T*_*TPI1*_*-P*_*TEF1*_*-tHMG1-T*_*CYC1*_, **SGH1**: BY-T20/pESC-Trp::SmCPS_*ent*_, **SGH2**: BY-T20/pESC-Trp::SmKS, **SGH3**: BY-T20/pESC-Trp::SmCPS_*ent*_/SmKS, **SGH4**: BY-T20/pESC-Trp::SmKS-SmCPS_*ent*_. (**B**) Extracted ion chromatograms showing the SmCPS_*ent*_ and SmKS products. **AtCPS** (*ent*-copalol, the dephosphorylated product of *ent*-CPP), **AtCPS + AtKS** (*ent*-kaurene). (**C**) The mass spectra of the SmCPS_*ent*_ and SmKS products. (**D**) Relative *ent*-kaurene contents in the yeast strains SGH3 and SGH4 based on their peak area ratio. The data represent the mean ± SD from three independent experiments.

Previously reported evidence suggested that SmCPS_*ent*_ and SmKS might be involved in the *S*. *miltiorrhiza* GA biosynthetic pathway^6^. To confirm the biochemical functions of SmCPS_*ent*_ and SmKS *in vivo*, the SmCPS_*ent*_ ORF and SmKS ORFs were ligated individually or in combination in the yeast expression vector pESC-Trp (Fig. 4A) and expressed in the yeast strain BY-T20 (provided by Prof. Xueli Zhang’s lab, Tianjin Institute of Industrial Biotechnology, Chinese Academy of Sciences, China). As expected, CPP was detected as the SmCPS_*ent*_ product in the products of the yeast strain SGH1 (BY-T20/pESC-Trp::SmCPS_*ent*_) compared with the product of the *A. thaliana* AtCPS using GGPP as the substrate. No CPP was found in the yeast strain carrying the empty pESC-Trp vector. Using the product of the *A. thaliana* AtKS as the authentic standard, *ent*-kaurene was detected as the SmKS product in the products of the yeast strain SGH3 (BY-T20/pESC-Trp::SmCPS_*ent*_/SmKS) but not SGH2 (BY-T20/pESC-Trp::SmKS), confirming that SmKS possessed monofunctional class I diTPS activity and catalyzed the formation of *ent*-kaurene using the SmCPS_*ent*_ product CPP as the substrate. Therefore, the absolute configuration of the SmCPS_*ent*_ product CPP was identified as an enantiomer (i.e., *ent*-CPP) (Fig. 4B).

Protein complexes have been reported to improve the efficiency of specific pathways by protecting substrates and intermediates from diffusion and degradation^16^. Zhou *et al.* reported that a recombinant strain containing the fused enzyme SmKSL1-SmCPS1 produced 2.8-fold more miltiradiene compared with another recombinant strain in which SmCPS1 and SmKSL1 were expressed separately^17^. Hence, we constructed the fused enzyme SmKS-SmCPS_*ent*_ in the yeast strain SGH4 (BY-T20/pESC-Trp::SmKS-SmCPS_*ent*_) using the RF cloning method, and the results showed that SGH4 produced approximately 4.25-fold more *ent*-kaurene than SGH3 (Fig. 4D).

### Recombinant expression and functional characterization of SmKO *in vivo*

**Figure 5:**
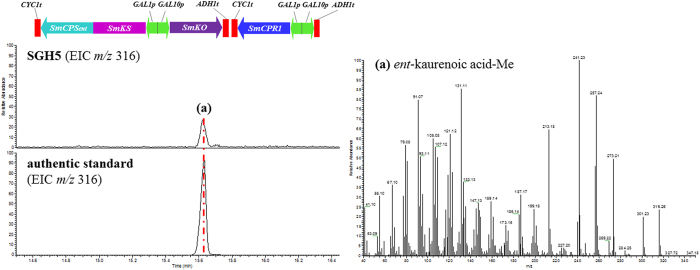
The product from BY-T20 yeast strains expressing the *S. miltiorrhiza SmKO*. SGH5: BY-T20/pESC-Trp::SmKS-SmCPS_*ent*_/SmKO + pESC-Leu::SmCPR1. Extracted ion chromatograms showing methyl ester derivatives of the SmKO product with its corresponding mass spectra.

As a strategy to characterize the biochemical function of SmKO *in vivo*, first we constructed the fused enzyme SmKS-SmCPS_*ent*_, which improved the *ent*-kaurene precursor supply as expected. Then, SmKO was coexpressed with the fused enzyme SmKS-SmCPS_*ent*_ and a NADPH-cytochrome P450 reductase (SmCPR1) in the yeast strain SGH5 (BY-T20/pESC-Trp::SmKS-SmCPS_*ent*_/SmKO + pESC-Leu::SmCPR1). After extraction and methylation, the *ent*-kaurenoic acid methyl ester was detected by a comparison with the methylated authentic standard (Sigma, USA) (Fig. 5). This result confirmed that *SmKO* encoded a functional *ent*-kaurene oxidase that was involved in the three-stage oxidation of *ent*-kaurene to *ent*-kaurenoic acid in the *S*. *miltiorrhiza* GA biosynthetic pathway.

## Discussion

We identified three consecutive enzymes (SmCPS_*ent*_, SmKS and SmKO) involved in the *S. miltiorrhiza* GA biosynthetic pathway. SmCPS_*ent*_ catalyzes the formation of *ent*-CPP from GGPP; then, SmKS converts *ent*-CPP to *ent*-kaurene. Subsequently, SmKO converts *ent*-kaurene to *ent*-kaurenoic acid via a three-stage oxidation reaction. *ent*-Kaurene biosynthesis was reported to be catalyzed by a one-to-one CPS/KS complex in which CPP could be channeled from CPS to the KS catalytic site^10^. Therefore, we fused SmCPS_*ent*_ and SmKS to obtain a close proximity between the active sites of the two consecutive enzymes. As expected, the fused enzyme SmKS-SmCPS_*ent*_ produced 4.25-fold more *ent*-kaurene than the separate expression of SmCPS_*ent*_ and SmKS in the yeast strain, suggesting that the protein fusion treatment was an efficient approach to improve the catalytic activity and enlarge the heterologous production of *ent*-kaurene. With an increased supply of the *ent*-kaurene precursor, SmKO catalyzed the formation of *ent*-kaurenoic acid. However, the intermediates *ent*-kaurenol and *ent*-kaurenal were not detected. One possible explanation is that the intermediates were unstable and were changed into other intermediates during the extraction process. The enzymes involved in the early steps of the GA biosynthetic pathway (i.e., CPS, KS, KO, and KAO) are primarily encoded by single genes, whereas those involved in the later steps (i.e., GA20ox, GA3ox, and GA2ox) are encoded by gene families^18^. The *SmCPS*_*ent*_, *SmKS* and *SmKO* genes are likely single copy genes responsible for GA biosynthesis in *S. miltiorrhiza*.

In addition to the identification and characterization of SmCPS_*ent*_, SmKS and SmKO, we provided insights into the genes encoding the enzymes involved in all steps of the GA biosynthetic pathway from GGPP to *ent*-kaurenoic acid. Our results provide a foundation for further characterization of the subsequent enzymes (i.e., SmKAO and the CYP88A subfamily) involved in the GA biosynthetic pathway using this yeast expression system. In plants, GA levels vary at different sites and during different development processes^19^. It is possible to control the GA levels by regulating the expression of these genes to acquire better growth of the *S. miltiorrhiza* roots and rhizomes, thereby improving the total yield of tanshinones per plant.

In conclusion, we functionally characterized three consecutive enzymes (SmCPS_*ent*_, SmKS and SmKO) involved in the GA biosynthetic pathway from GGPP to *ent*-kaurenoic acid, thereby laying the foundation for further characterization of GA biosynthesis. Based on these results, we could regulate the expression of all genes involved in the GA biosynthetic pathway to acquire better growth and an increased accumulation of the bioactive tanshinones involved in the *S. miltiorrhiza* developmental processes. Protein fusion is an applicable and efficient approach that can be used to direct metabolic flux to the bioactive diterpenoid tanshinones pathway for the heterologous production of isoprenoids in microbial cell factories.

## Methods

### RNA isolation and cDNA cloning

Hairy roots were induced from the *S. miltiorrhiza* leaf explants under the mediation of *A*. *rhizogenes* strain ACCC10060 as described previously^20^ and maintained in 6,7-V liquid medium^21^ at 25 °C on a gyratory shaker (80 rpm) in the dark. Total RNA was extracted using the TRIzol reagent (Invitrogen, Carlsbad, CA, USA). The 5′ and 3′ ends of the targeted *SmCPS*_*ent*_ and *SmKS* genes were cloned by RACE (Invitrogen) according to the manufacturer’s directions using the corresponding *S. miltiorrhiza* genome sequences released by the National Center for Biotechnology Information (NCBI)^22^. The primer sequences are shown in Supplementary Table 1. An aliquot (1 μg) of the total RNA was used to synthesize the first strand cDNA according to the PrimeScript 1^st^ Strand cDNA Synthesis Kit (Takara Bio, Dalian, China) manufacturer’s protocol. The full-length cDNA for each ORF was cloned using the PrimeSTAR DNA polymerase (Takara Bio). The PCR products were purified and cloned into the pEASY-T3 cloning vector (TransGen Biotech, Beijing, China), transformed into *Escherichia coli* Trans5α cells (TransGen Biotech), and then cultured in Luria-Bertani (LB) medium at 37 °C in the dark. Positive clones were sequenced. The full-length cDNA of *SmKO* was cloned previously^23^.

### Bioinformatics analysis

The *SmCPS*_*ent*_, *SmKS* and *SmKO* sequences were confirmed at NCBI (http://www.ncbi.nlm.nih.gov/). The open reading frames (ORFs) and deduced amino acid sequences were analyzed using the online tool ORF Finder (http://www.ncbi.nlm.nih.gov/gorf/gorf.html) and the ExPASy online tool (http://web.expasy.org/translate/), respectively. The ChloroP 1.1 Server (http://www.cbs.dtu.dk/services/ChloroP/) was used to predict chloroplast transit peptides. The sequences of the *SmCPS*_*ent*_, *SmKS* and *SmKO* as well as other corresponding sequences downloaded from GenBank were aligned using the DNAMAN program, and the phylogenetic trees for *SmCPS*_*ent*_, *SmKS* and *SmKO* were constructed using sequences from other plants (Supplementary Table 2) using the neighbor-joining method in MEGA5.1 24.

### Recombinant expression and functional characterization of SmCPS_*ent*_ and SmKS

The SmCPS_*ent*_ and SmKS ORFs (alone or in combination) were subcloned into the yeast epitope-tagging vector pESC-Trp under the control of the GAL1 or GAL10 inducible promoter (Agilent Technologies, USA) via digestion by the corresponding restriction endonucleases. The resulting constructs were verified by complete gene sequencing and then transformed into the yeast strain BY-T20 (BY4742, *ΔTrp1*, *Trp1::HIS3-P*_*PGK1*_*-BTS1/ERG20-T*_*ADH1*_*-P*_*TDH3*_*-SaGGPS-T*_*TPI1*_*-P*_*TEF1*_*-tHMG1-T*_*CYC1*_, provided by Prof. Xueli Zhang’s lab, Tianjin Institute of Industrial Biotechnology, Chinese Academy of Sciences, China)^25^,^26^,^27^. Then, the recombinant strains SGH1 (containing the plasmid pESC-Trp::SmCPS_*ent*_), SGH2 (containing the plasmid pESC-Trp::SmKS), and SGH3 (containing the plasmid pESC-Trp::SmCPS_*ent*_/SmKS) were selected on synthetic dropin medium -Trp-His (SD-Trp-His) containing 20 g/L glucose and grown at 30 °C for 2–3 d. Single transformed yeast colonies were grown in SD-Trp-His liquid medium supplemented with 20 g/L glucose at 30 °C for approximately 2 d. The yeast cells were pelleted and resuspended in 100 mL of SD-Trp-His liquid induction medium supplemented with 20 g/L galactose and grown at 30 °C for 3 d. Finally, the induced yeast cells were extracted three times with an equal volume of hexane. The organic fractions were pooled and dried using a Nitrogen Evaporator (Baojingkeji, Henan, China). The dried samples were dissolved in 100 μL of hexane for GC-MS analysis as described previously^28^. To confirm the products of these strains, the identified products *ent*-CPP and *ent*-kaurene of *A. thaliana* AtCPS and AtKS were used as the authentic standards^29^,^30^. The detailed protocols for the constructions of the recombinant plasmids and strains and the recombinant expression and enzymatic assay for AtCPS and AtKS are described in the Supplementary Methods.

### Construction of the module producing the fused protein SmKS-SmCPS_*ent*_ and the functional characterization of SmKO

To prepare the module producing the fused protein SmKS-SmCPS_*ent*_, a restriction-free (RF) cloning method was used^31^. The genes encoding the fusion enzyme were constructed by inserting a widely used GGGS linker encoded by a “GGT GGT GGT TCT” sequence between the two corresponding genes^32^,^33^. The recombinant plasmid pESC-Trp::SmKS-SmCPS_*ent*_ was transformed into the yeast strain BY-T20 to generate SGH4 and induced with D-galactose as described above. Then, the products of SGH4 were analyzed by GC-MS. The detailed protocols for the RF cloning are described in the Supplementary Methods.

The ORF region of SmKO was ligated into the recombinant plasmid pESC-Trp::SmKS-SmCPS_*ent*_ as described above and then transformed into the yeast strain BY-T20 with another recombinant plasmid pESC-Leu::SmCPR1 (SmCPR1, *S. miltiorrhiza* cytochrome P450 reductase^8^). The recombinant strain SGH5 (containing the plasmids pESC-Trp::SmKS-SmCPS_*ent*_/SmKO and pESC-Leu::SmCPR1) was induced with D-galactose and extracted once with an equal volume of hexane and twice with an equal volume of ethyl acetate. The organic fractions were pooled and dried and then dissolved in 50 μL of methanol and methylated with approximately 200 μL of (trimethylsilyl)diazomethane (Aladdin Industrial Inc., Shanghai, China). The methylated samples were redried and then dissolved in 100 μL of ethyl acetate for GC-MS using a Thermo TRACE 1310/TSQ 8000 gas chromatograph (splitless; injector temperature 250 °C) with a DB-5 ms (30 m × 0.25 mm × 0.25 μm) capillary column. The GC conditions were the same as those described previously^34^.

## Additional Information

**How to cite this article**: Su, P. *et al.* Functional characterization of *ent*-copalyl diphosphate synthase, kaurene synthase and kaurene oxidase in the *Salvia miltiorrhiza* gibberellin biosynthetic pathway. *Sci. Rep.* **6**, 23057; doi: 10.1038/srep23057 (2016).

## Supplementary Material

Supplementary Information

## Figures and Tables

**Figure 1:**
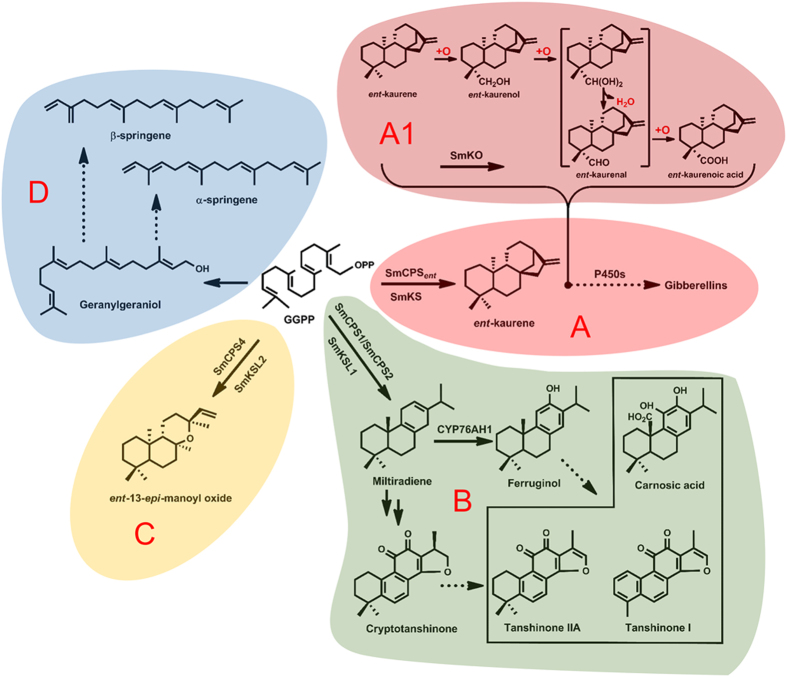
Four different diterpenoid biosynthetic pathways in *S. miltiorrhiza*. (**A**) Gibberellin biosynthetic pathway. (**A1**) Proposed mechanism for SmKO conversion of *ent*-kaurene into *ent*-kaurenoic acid^35^. (**B**) Tanshinone biosynthetic pathway. (**C**,**D**) Unknown diterpenoid biosynthetic pathways.
