# Supplementary material for: Functional characterization of ent-copalyl diphosphate synthase, kaurene synthase and kaurene oxidase in the Salvia miltiorrhiza gibberellin biosynthetic pathway
Source: Sci Rep. 2016 Mar 14;6:23057. doi: 10.1038/srep23057 (PMC4789781; doi:10.1038/srep23057)
Supplement: Supplementary Information [file srep23057-s1.doc]

Functional characterization of *ent*-copalyl diphosphate synthase, kaurene synthase and kaurene oxidase in the *Salvia miltiorrhiza* gibberellin biosynthetic pathway

Ping Su^1,2†^, Yuru Tong^1,2†^, Qiqing Cheng^1,2,3^, Yating Hu^4^, Meng Zhang^1^, Jian Yang^2^, Zhongqiu Teng^2^, Wei Gao^1*^, Luqi Huang^2*^

^1^School of Traditional Chinese Medicine, Capital Medical University, Beijing, China

^2^State Key Laboratory Breeding Base of Dao-di Herbs, National Resource Center for Chinese Materia Medica, China Academy of Chinese Medical Sciences, Beijing, China

^3^State Key Laboratory of Quality Research in Chinese Medicine, Macau University of Science and Technology, Avenida Wai Long, Taipa, Macau, China

^4^Department of Chemical and Biological Engineering, Chalmers University of Technology, Kemivägen 10, SE-41296 Göteborg, Sweden

^†^These authors contributed equally to this work

E-mail: [weigao@ccmu.edu.cn](mailto:weigao@ccmu.edu.cn)

Tel: +86-10-8391-1633

Fax: +86-10-8391-1627

E-mail: [huangluqi01@126.com](mailto:huangluqi01@126.com)

Tel: +86-10-6401-4411-2955

Fax: +86-10-6401-3996

**Supplementary Methods**

**Construction of strains and plasmids**

All the PCR primers are listed in supplementary Table 1.

The PCR product of SmCPS*_ent_* ORF was digested with *BamH*I and *Apa*I (NEB, New England Biolabs, Beverly, MA, USA), and ligated into pESC-Trp, generating the plasmid pESC-Trp::SmCPS*_ent_*. The same operation was applied to the PCR product of SmKS ORF, to yield the plasmid pESC-Trp::SmKS using *Not*I and *Spe*I sites, then SmCPS*_ent_* ORF digested with *BamH*I and *Apa*I was introduced into the plasmid pESC-Trp::SmKS, generating the plasmid pESC-Trp::SmCPS*_ent_*/SmKS. And the PCR product of SmCPR1 ORF was subcloned into the *BamH*I and *Sal*I sites of pESC-Leu, yielding the plasmid pESC-Leu::SmCPR1.

To create the plasmid pESC-Trp::SmKS-SmCPS*_ent_*/SmKO, the module SmKS-SmCPS*_ent_* was firstly constructed using a restriction-free (RF) cloning method^1^ (The details were described later), and inserted into the *BamH*I and *Apa*I sites of pESC-Trp, yielding the plasmid pESC-Trp::SmKS-SmCPS*_ent_*. Then SmKO ORF digested with *Not*I and *Cla*I was introduced into the constructed pESC-Trp::SmKS, creating the plasmid pESC-Trp::SmKS-SmCPS*_ent_*/SmKO.

**Recombinant expression and enzymatic assay of AtCPS and AtKS**

Based on the full length cDNA sequences of *Arabidopsis thaliana AtCPS* and *AtKS* (GenBank accession number: U11034 and NM_106594), the primers were designed to subcloned the ORF of *AtCPS* and *AtKS* into pMAL-c2X expression vecter (NEB), respectively, then the recombinant plasmids pMAL-c2X::AtCPS and pMAL-c2X::AtKS were separately transformed into the TransB(DE3) strain (TransGen Biotech, Beijing, China). Single colonies of freshly transformed cells were cultured in 1mL Luria-Bertani (LB) medium at 37 °C overnight (250 rpm), with 100 mg/L ampicillin. The initial cultures was then added into 100 mL LB medium (containing 100 mg/L ampicillin), and grown to A_600_ approximately 0.6-0.8 (37 °C, 250 rpm, 2-3 h). These cultures were transferred to 16 °C (250 rpm) for 8 h induction with 0.4 mM IPTG (Sigma, USA). The cells were harvested by centrifugation (3000 g, 20 min, 4 °C) and stored at -80 °C.

The harvested cells were resuspended in 5 mL HEPES buffer (50 mM HEPES, pH 7.2, 100 mM KCl, 7.5 mM MgCl_2_, 5% glycerol, and 5 mM DTT)^2^, and lysed by sonication for 3 min (lysed for 10 s, paused for 10 s, 6 times). The resulting lysates were centrifuged at 12,000 rpm (4 °C) for 30 min, and the recombinant protein was partially purified using Amicon Ultra-15 centrifugal filter unit with Ultracel-30 membrane (Merck Millipore, Germany), according to the manufacturer's instructions. Enzymatic analysis was carried out in HEPES buffer with the recombinant protein of AtCPS, and initiated by the addition of GGPP to 200 µM for a final volume of 0.2 mL. After incubating at room temperature for 2 h, the reactions were extracted three times with 0.5 mL hexane and the residual organic solvent was removed using a Nitrogen Evaporators (Baojingkeji, Henan, China) to enable enzymatic dephosphorylation by calf intestinal phosphatase (NEB), proceeding at 37 °C for 4 h. The dephosphorylated compounds were extracted with hexane (3 × 0.5 mL), then dried using a Nitrogen Evaporators and redissolved in 100 µL of hexane for GC-MS analysis described previously^3^. For in vitro assay of AtKS, GGPP was incubated with the recombinant protein of AtCPS for 2 h prior to adding equivalent volume of the recombinant protein of AtKS, along with MgCl_2_ to a final concentration of 10 mM, and the reactions incubated overnight at room temperature before extraction with hexanes (3 × 0.5 mL) and subsequent GC-MS analysis.

**Construction of the module producing the fused protein SmKS-SmCPS*_ent_***

Two primers, SmKS-SmCPS*_ent_*-F and SmKS-SmCPS*_ent_*-R for RF cloning, were listed in supplementary Table 1. The RF reaction I was performed within a total volume of 50 µL with 20 ng pEASY-T3-SmKS as templet using PrimeSTAR DNA polymerase, and the products were used as the mega-primer in the RF reaction II, which contained 100 ng pEASY-T3::SmCPS*_ent_*, 500 ng RF reaction I products, 10 µL 5 × PrimeSTAR GXL buffer, 5 µL dNTP Mixture (2.5 mM) and 1.5 µL PrimeSTAR DNA polymerase. The cycling parameters consisted of an initial denaturation at 94 °C for 5 min, 35 cycles of 94 °C for 30 s, 58 °C for 1 min and 72 °C for 8 min, and a final extension step at 72 °C for 10 min. Reaction mix (10 µL) was transferred into a new tube and treated with 1 µL *Dpn*I restriction endonuclease (Takara Bio, Dalian, China) at 37 °C for 4 h to digest the methylated parental plasmid. After that, 5 µL digestion mixture were transformed into *E*. *coli* Trans5α cells, and cultured in LB medium at 37 °C in the dark. Positive clones were identified and cultured in LB medium for abstracting the recombinant plasmid, pEASY-T3::SmKS-SmCPS*_ent_*.

1. van den Ent, F. & Lowe, J. RF cloning: a restriction-free method for inserting target genes into plasmids. *Journal of Biochemical and Biophysical Methods* **67**, 67-74 (2006).
2. Xu, MM., Hillwig, M. L., Prisic, S., Coates, R. M. & Peters, R. J. Functional identification of rice *syn*-copalyl diphosphate synthase and its role in initiating biosynthesis of diterpenoid phytoalexin/allelopathic natural products. *The Plant Journal* **39**, 309-318 (2004).
3. Zhang, M. *et al*. Identification of geranylgeranyl diphosphate synthase genes from *Tripterygium wilfordii*. *Plant Cell Reports* doi:10.1007/s00299-015-1860-3 (2015).

**Supplementary Table 1. Primers used in this study**

| Primer Name | Primer sequence (5`→3`) |
| --- | --- |
| **RACE-PCR** | |
| SmCPS*_ent_*-5’ | CCCTTTGTGGGTTTTGTCGGGATGTA |
| SmCPS*_ent_*-3’ | CAAGGACTTTGGTGGGGACATTACTCAG |
| SmKS-5’ | GCAAAGGGTGATGGTGGGGACG |
| SmKS-3’ | AGAGTGTCCTTCCGAAGCCGTGC |
| **ORF-PCR** | |
| SmCPS*_ent_*-*BamH*I-F | CGCGGATCCGATGCCTCTCGCTTCCAATCC |
| SmCPS*_ent_*-*Apa*I-R | TCGGGGCCCAATTGTACTCTTTCAAAGAGTACTTTTGC |
| SmKS-*Not*I-F | ATTTGCGGCCGCAATGGCGCTTCCTCTCTCCAC |
| SmKS-*Spe*I-R | GGACTAGTGCATCATGAAGCTTTGATAATCCTGCT |
| SmKO-*Not*I-F | ATTTGCGGCCGCAATGGATACACTGCTGAGTCTTCAAGC |
| SmKO-*Cla*I-R | CCCATCGATACTGAGACACACACACGATCCCTTAGTC |
| SmCPR1-*BamH*I-F | CGCGGATCCGATGGAACCCTCGTCGAAGAAGCTC |
| SmCPR1-*Sal*I-R | CGCGTCGACCCATACATCGCGCAAGTACCTTCCCG |
| SmKS-SmCPS*_ent_*-*BamH*I-F | CGCGGATCCGATGGCGCTTCCTCTCTCCAC |
| SmKS-SmCPS*_ent_*-*Apa*I-R | TCGGGGCCCAATTGTACTCTTTCAAAGAGTACTTTTGC |
| AtCPS-*BamH*I-F | CGCGGATCCATGTCTCTTCAGTATCATGTTCTAAACTCC |
| AtCPS-*Pst*I-R | GCACTGCAGCTAGACTTTTTGAAACAAGACTTTGGAG |
| AtKS-*BamH*I-F | CGCGGATCCATGTCTATCAACCTTCGCTCCTC |
| AtKS-*Sal*I-R | CGCGTCGACTCAAGTTAAAGATTCTTCCTGTAAGC |
| **RF-PCR** | |
| SmKS-SmCPS*_ent_*-F | GTGATTGGGATCGCCCTTCGCGGATCCGATGGCGCTTCCTCTCTCCACTTGTCTC |
| SmKS-SmCPS*_ent_*-R | GACGGGATTGGAAGCGAGAGGCATAGAACCACCACCATCATGAAGCTTTGATAATCCTG |

* The underlined bases represent the restriction sites used for cloning. F, forward; R, reverse.

**Supplementary Table 2. Descriptions of** **diTPSs and CYP enzymes used in Fig 2**

| **Protein** | **Species** | **GenBank Accession** | **Annotation** |
| --- | --- | --- | --- |
| **CPSs** | | | |
| SdCPS | *Scoparia dulcis* | BAB03594 | copalyl diphosphate |
| SmCPS5 | *Salvia miltiorrhiza* f. alba | AHJ59324 | *ent*-copalyl diphosphate synthase |
| **SmCPS*_ent_*** | ***Salvia miltiorrhiza*** | **ALX18648** | ***ent*-****copalyl diphosphate synthase** |
| ApCPS | *Andrographis paniculata* | AEM00024 | *ent*-copalyl diphosphate synthase |
| CaCPS | *Coffea arabica* | ACQ99373 | copalyl diphosphate synthase |
| PcCPS | *Pyrus communis* | AGF25267 | copalyl diphosphate synthase |
| CmCPS | *Castanea mollissima* | AEF32082 | copalyl diphosphate synthase |
| TcCPS | *Theobroma cacao* | XP_007050589 | copalyl diphosphate synthase |
| ZmCPS | *Zea mays* | AAT70084 | *ent*-copalyl diphosphate synthase |
| TaCPS | *Triticum aestivum* | BAH56558 | *ent*-copalyl diphosphate synthase |
| OsCPS | *Oryza sativa* Indica Group | AAT11021 | *ent*-copalyl diphosphate synthase |
| PsCPS | *Picea sitchensis* | ADB55709 | *ent*-copalyl diphosphate synthase |
| PgCPS | *Picea glauca* | ADB55707 | *ent*-copalyl diphosphate synthase |
| **KSs** | | | |
| MdKS | *Malus domestica* | AFG18184 | *ent*-kaurene synthase |
| PcKS | *Pyrus communis* | AEN74904 | *ent*-kaurene synthase |
| CmKS | *Castanea mollissima* | AEF32083 | *ent*-kaurene synthase |
| GhKS | *Gossypium hirsutum* | AIY27526 | *ent*-kaurene synthase |
| SdKS | *Scoparia dulcis* | AEF33360 | *ent*-kaurene synthase |
| PbKS | *Plectranthus barbatus* | AGN70881 | *ent*-kaurene synthase |
| SmKSL2 | *Salvia miltiorrhiza* f.alba | AHJ59325 | kaurene synthase |
| **SmKS** | ***Salvia miltiorrhiza*** | **ALX18649** | **kaurene synthase** |
| OsKS | *Oryza sativa* Japonica Group | AAQ72560 | *ent*-kaurene synthase |
| TaKS | *Triticum aestivum* | ADZ55290 | *ent*-kaurene synthase |
| PsKS | *Picea sitchensis* | ADB55710 | (-)-*ent*-kaurene synthase |
| PgKS | *Picea glauca* | ACY25275 | (-)-*ent*-kaurene synthase |
| **KOs** | | | |
| CaKO | *Coffea arabica* | ACQ99375 | *ent*-kaurene oxidase |
| CsKO | *Cucumis sativus* | NP_001267703 | *ent*-kaurene oxidase |
| McKO | *Momordica charantia* | ADE06669 | *ent*-kaurene oxidase |
| MtKO | *Medicago truncatula* | XP_003637273 | *ent*-kaurene oxidase |
| PsKO | *Pisum sativum* | AAP69988 | *ent*-kaurene oxidase |
| GsKO | *Glycine soja* | KHN31869 | *ent*-kaurene oxidase |
| **SmKO** | ***Salvia miltiorrhiza*** | **AJF93403** | ***ent*-kaurene oxidase** |
| MnKO | *Morus notabilis* | XP_010089925 | *ent*-kaurene oxidase |
| PcKO | *Pyrus communis* | AEK01241 | *ent*-kaurene oxidase |
| AtKO | *Arabidopsis thaliana* | NP_197962 | *ent*-kaurene oxidase |
| JcKO | *Jatropha curcas* | NP_001292955 | *ent*-kaurene oxidase |
| PtKO | *Populus trichocarpa* | XP_006386514 | *ent*-kaurene oxidase |
| OsKO | *Oryza sativa* Japonica Group | AAT81230 | *ent*-kaurene oxidase |
| TaKO | *Triticum aestivum* | ADZ55286 | *ent*-kaurene oxidase |
| ZmKO | *Zea mays* | ACG38493 | *ent*-kaurene oxidase |
| PpKO | *Physcomitrella patens* | BAK19917 | *ent*-kaurene oxidase |

**
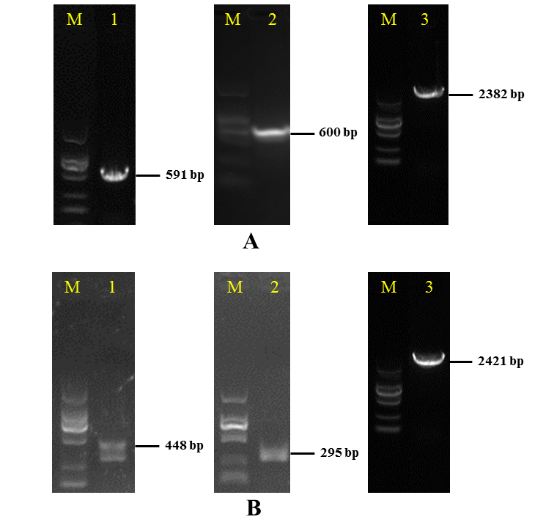
**

**Supplementary Figure 1. Results of agarose gel electrophoresis.** M: DL2000 DNA Marker (Takara Bio), 1: 5`RACE. 2: 3`RACE. 3: ORF. A: *SmCPS_ent_*, B: *SmKS*.
